# Supplementary material for: End Sequence Analysis Toolkit (ESAT) expands the extractable information from single-cell RNA-seq data
Source: Genome Res. 2016 Oct;26(10):1397–410. doi: 10.1101/gr.207902.116 (PMC5052061; doi:10.1101/gr.207902.116)
Supplement: Supplemental Material [file supp_gr.207902.116_Supp_Materials.tar.gz › ESAT-esat_v0.1/doc/html/classumms_1_1core_1_1model_1_1_j_c_s_alignment_model_1_1_filtered_iterator.html]

ESAT: umms.core.model.JCSAlignmentModel.FilteredIterator Class Reference


ESAT


- Main Page
- Related Pages
- Packages
- Classes
- Files

- Class List
- Class Index
- Class Hierarchy
- Class Members

All Classes Namespaces Files Functions Variables Enumerator Pages

- umms
- core
- model
- JCSAlignmentModel
- FilteredIterator

Public Member Functions |
List of all members

umms.core.model.JCSAlignmentModel.FilteredIterator Class Reference

Inheritance diagram for umms.core.model.JCSAlignmentModel.FilteredIterator:

[legend]

Collaboration diagram for umms.core.model.JCSAlignmentModel.FilteredIterator:

[legend]

|  |  |
| --- | --- |
| Public Member Functions | |
|  | FilteredIterator (CloseableIterator< AlignmentCount > overlappers, Annotation region, CoordinateSpace cs, boolean fullyContained) |
|  | |
|  | FilteredIterator (CloseableIterator< AlignmentCount > reads) |
|  | |
| boolean | hasNext () |
|  | |
| AlignmentCount | next () |
|  | |
| void | remove () |
|  | |
| void | close () |
|  | |

## Detailed Description

This class will represent an iterator over the filtered reads To make it efficient it will need to precompute the region in coordinate space It will also need to know the next element to compute the hasNext and getNext() functions

Author
:   mguttman

## Constructor & Destructor Documentation

|  |  |  |  |
| --- | --- | --- | --- |
| umms.core.model.JCSAlignmentModel.FilteredIterator.FilteredIterator | ( | CloseableIterator< AlignmentCount > | *overlappers*, |
|  |  | Annotation | *region*, |
|  |  | CoordinateSpace | *cs*, |
|  |  | boolean | *fullyContained* |
|  | ) |  |  |

Here is the call graph for this function:

|  |  |  |  |  |  |
| --- | --- | --- | --- | --- | --- |
| umms.core.model.JCSAlignmentModel.FilteredIterator.FilteredIterator | ( | CloseableIterator< AlignmentCount > | *reads* | ) |  |

This is the constructor for all reads other parms not meaningful

Parameters
:   |  |  |
    | --- | --- |
    | reads |  |

Here is the call graph for this function:

## Member Function Documentation

|  |  |  |  |  |
| --- | --- | --- | --- | --- |
| void umms.core.model.JCSAlignmentModel.FilteredIterator.close | ( |  | ) |  |

|  |  |  |  |  |
| --- | --- | --- | --- | --- |
| boolean umms.core.model.JCSAlignmentModel.FilteredIterator.hasNext | ( |  | ) |  |

Here is the call graph for this function:

Here is the caller graph for this function:

|  |  |  |  |  |
| --- | --- | --- | --- | --- |
| AlignmentCount umms.core.model.JCSAlignmentModel.FilteredIterator.next | ( |  | ) |  |

Here is the call graph for this function:

Here is the caller graph for this function:

|  |  |  |  |  |
| --- | --- | --- | --- | --- |
| void umms.core.model.JCSAlignmentModel.FilteredIterator.remove | ( |  | ) |  |

---

The documentation for this class was generated from the following file:

- java/umms/core/model/JCSAlignmentModel.java


---

Generated on Fri Aug 15 2014 15:11:11 for ESAT by  

 1.8.7
